# Disease burden attributable to respiratory syncytial virus outbreaks in long-term care

**DOI:** 10.14745/ccdr.v50i12a03

**Published:** 2024-01-01

**Authors:** Christina Ferrante, Christina Bancej, Nicole Atchessi

**Affiliations:** 1 Centre for Emerging and Respiratory Infections and Pandemic Preparedness, Public Health Agency of Canada, Ottawa, ON

**Keywords:** respiratory syncytial virus, RSV, burden, long-term care, outbreak

## Abstract

**Background:**

Respiratory syncytial virus (RSV) disease burden is significant among children; however, RSV can also cause excess morbidity and mortality among older adults. Populations in long-term care homes (LTCHs) may be at greater risk of exposure and increased infection severity. The objectives of this article are to identify evidence regarding disease burden and outcome severity attributable to RSV outbreaks among residents and staff in LTCHs; and to highlight reported population and outbreak characteristics.

**Methods:**

All types of evidence were eligible for inclusion. Data utilized by included studies was between the end of the 2010 H1N1 influenza pandemic and the beginning of the coronavirus disease 2019 (COVID-19) pandemic. Evidence from the following countries was considered: G7, the European Union, Australia and New Zealand. A total of 167 articles were identified; 58 full texts were analyzed and four sources of evidence were eligible for inclusion. Data related to population characteristics, outbreak type and resident and staff outcomes were manually charted.

**Results:**

There is a paucity of evidence sources pertaining to RSV outbreak burden among residents and staff in LTCHs. Outbreak duration ranged from 13 to 21 days. For each outbreak, 4–7 residents had confirmed RSV infection. Attack rates ranged from 12% to 38%. A spectrum of disease attributable to RSV outbreaks in LTCHs was identified, ranging from mild cold-like symptoms to death.

**Conclusion:**

Integration of RSV into existing respiratory pathogen surveillance programs is important to characterize susceptibility, transmissibility and virulence of RSV in at-risk populations. There is a need for public health organizations to publish the findings from outbreak investigations to provide evidence to inform RSV outbreak prevention and response in LTCH settings.

## Introduction

Respiratory syncytial virus (RSV) is a pathogen responsible for a significant proportion of lower respiratory tract illnesses worldwide ((1–3)). It is mostly associated with causing disease burden among infants and young children; however, a significant burden can be placed on older and at-risk adults by RSV, as it is considered one of the most significant causes of excess morbidity and mortality among older adults ((4–8)). Respiratory syncytial virus may lead to complications and severe outcomes like those caused by seasonal influenza infection among older adults ((9–11)).

Long-term care (LTC) residents and staff spend significant time in congregated indoors settings where respiratory outbreaks are commonplace ((12)). Long-term care residents, who tend to be 80 years old on average, may be at increased risk of heightened respiratory infection severity—including severe symptoms, hospitalization, or mortality ((13–15)). Considering the growing proportion of older adults and projected increased demand for LTC services and staff, LTC populations might require additional attention to prevent and mitigate the consequences of LTC outbreaks of RSV ((16–18)).

Disease burden due to RSV outbreaks among LTC populations can be mitigated; however, for prevention and response to be population specific and most effective, these interventions need to be evidence-informed. Understanding the extent of currently available evidence and current knowledge gaps could help to inform subsequent research and public health activities with the goal of minimizing the burden in long-term care homes (LTCHs) from RSV outbreaks. Currently, little evidence synthesis on RSV outbreak burden among residents and staff in LTC settings is available, which makes current gaps in the literature challenging to identify. Some recently published reviews on RSV outbreaks and disease burden among older adults and in LTC exist ((4,15,19)); however, these reviews are not specific to both LTC populations and RSV outbreaks. This is the first scoping review to synthesize available evidence related to RSV outbreak burden among residents and staff in LTCHs using more recently published literature from 2010 to 2020. The objectives of this review are a) to understand the extent of the evidence regarding disease burden attributable to RSV outbreaks in LTCHs, among both residents and staff; b) to highlight reported population and outbreak characteristics; and c) to highlight RSV outcome severity among residents and staff members.

## Methods

### Eligibility criteria

Eligibility criteria were determined before screening and review of identified sources (**Table 1**). Studies were eligible for inclusion if they were published in English or French and utilized data collected between the end of the H1N1 pandemic (2010) and the start of the coronavirus disease 2019 (COVID-19) pandemic (2020). The identification of a viral pathogen that is etiologically responsible for respiratory illness became more common around the time of the H1N1 pandemic, with the inclusion of other viral pathogens, including RSV, on routine multiplex polymerase chain reaction (PCR) tests. Therefore, this date range was chosen to include existing studies from the period where viral identification became more widespread, as well as to identify relevant literature that reflects more recent RSV dynamics in LTCHs. Sources were included if they discussed outbreaks of RSV in LTCHs in any G7 country (Canada, France, Germany, Italy, Japan, the United Kingdom, the United States, and the European Union—which is a non-enumerated member of the G7), Australia or New Zealand. These locations were chosen because their seasonal patterns of RSV outbreaks and culture of health care use and access are like Canada’s. Sources were included if the reported outbreaks occurred in LTC settings, which were defined as residential institutions in which primarily older adults receive care. Mixed outbreaks were included in the review if at least one case of RSV was detected in the outbreak. Studies were not excluded based on the type of diagnostic or confirmatory testing that was used. Outbreaks involving only residents, only staff or both residents and staff were considered for inclusion.

**Table 1:** Inclusion and exclusion criteria

Inclusion criteria	Exclusion criteria
I*1: Studies published and used data that were collected between April 2010 and March 2020.	E*1: Excluded studies that were published in 2010–2020 but using data collected during the H1N1 influenza and/or COVID-19 pandemics.
I*2: Published in English or French.	E*2: Excluded review studies that did not provide discussion regarding RSV disease burden in any of the identified populations as these studies will not provide relevant information to answer the research questions.
I*3: Study included or assessed data from G7 countries, Australia or New Zealand.	-
I*4: Population under study includes: older adults in LTC and adults working in LTC.	-
I*5: Studies that assessed the epidemiology (incidence, severity, mortality) of RSV outbreaks.	-
I*6: Studies that assessed the clinical epidemiology (presentation, course, dynamics, and severity) of RSV. In scope: clinical severity outcomes are hospitalization, ICU admission, death, duration of outbreak(s), severe symptoms.	-
I*7: Studies that assessed RSV disease burden at a population/outbreak level.	-

Abbreviations: COVID-19, coronavirus disease 2019, ICU, intensive care unit; LTC, long-term care; RSV, respiratory syncytial virus; -, there were no items to list in the cell

### Search strategy

Four databases were searched to identify potentially relevant evidence sources: MEDLINE, Embase, Global Health, and Scopus. The search strategy was developed with input from all authors. The literature search was conducted by the Health Canada Library. Keywords used for the literature search identified the setting, population, and outcomes of interest, including “LTC outbreak”, “respiratory syncytial virus”, “nursing home”, “older adult*”, “hospital*”, “mortalit*” and “respiratory infection”. A sample search of one database is presented in **Appendix** (**Table A1**). Due to time constraints, a grey literature search was not conducted as part of this review.

### Selection of evidence sources

**Figure 1:**
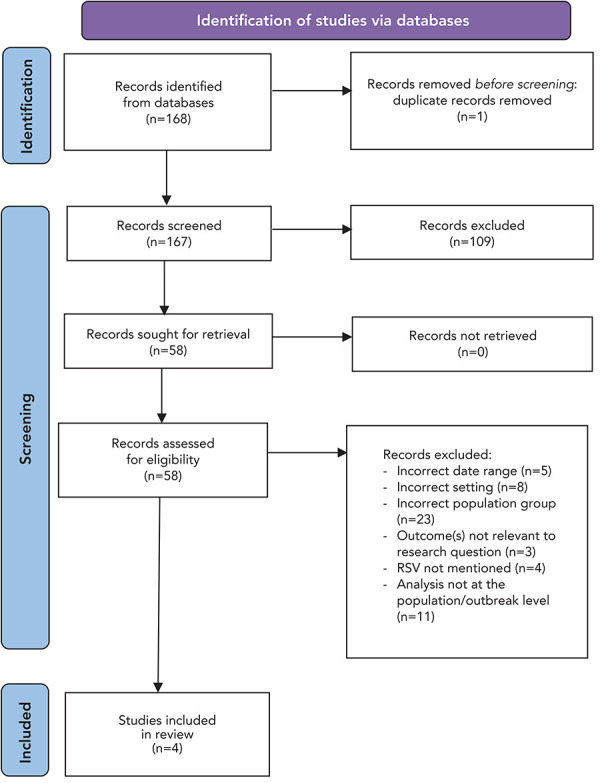
Selection process for included studies based on literature searches^a^ Abbreviation: RSV, respiratory syncytial virus ^a^ Adapted from ((20))

After removal of duplicates, 167 articles were considered for inclusion. References were imported into Zotero, a reference management system. Covidence, a screening and data extraction tool, was used during the screening process. Due to time constraints for the completion of this study, screening was conducted by a single reviewer. The first stage involved screening the titles and abstracts of all 167 articles. Inclusion and exclusion criteria were applied to determine whether the article would move onto the second round of screening. The second round involved the full-text review of 58 articles. Inclusion and exclusion criteria were used to determine if the source was eligible for inclusion in the review (Table 1). After full-text review, four articles met inclusion criteria, and were included in this review (**Figure 1**).

Data charting was conducted by a single reviewer using an online form developed using Covidence; however, data charting criteria were developed with input from all authors.

### Synthesis of results and quality assessment

Population characteristics (age, gender, type and number of LTC settings affected per outbreak) and outbreak characteristics (pathogens involved, outbreak duration, symptom and outcome severity, attack rates and number of residents and staff cases) were extracted, grouped and analyzed. Individual characteristics of each article (data collection dates, study population and location) were also extracted, grouped and analyzed.

## Results

The four articles included in the scoping review were published and used data collected between 2013 and 2017. **Table 2** describes the characteristics of each included article (n=4) for which data were charted. The studies were conducted in four different countries: Japan; Slovenia; the Netherlands; and the United States. Three articles are case series and one is a prospective study. The sample sizes across all included articles ranged from 10 to 99 residents, and zero to 42 staff members. All were primary sources of evidence.

**Table 2:** Characteristics of included sources of evidence (n=4)

Author, citation	Study title	Journal	Publication type	Publication date	Study location (country)	Sample size	Study purpose	Study outcome(s)
Doi *et al.*, ((21))	An outbreak of acute respiratory infections due to human respiratory syncytial virus in a nursing home for the elderly in Ibaraki, Japan, 2014	*Japan Journal Infectious Diseases*	Case series	2014	Japan	99 residents	To report the molecular epidemiological analysis of an outbreak of RSV in a nursing home.	Genetic sequences showed RSV-B outbreak in nursing home with 24 infected and 5 of 24 residents received a pneumonia diagnosis.
Meijer *et al.*, ((22))	Outbreak of respiratory syncytial virus infections in a nursing home and possible sources of introduction: The Netherlands, winter 2012/2013	*The American Geriatric Society*	Case series	2013	The Netherlands	10 residents	To describe an outbreak of RSV in a nursing home and to identify possible sources of introduction.	Four RSV positive cases among residents detected during the outbreak, all experienced mild symptoms and recovered within 2 weeks of illness onset.
Spires *et al.*, ((23))	Paramyxovirus Outbreak in a Long-Term Care Facility: The Challenges of Implementing Infection Control Practices in a Congregate Setting	*Infection Control and Hospital Epidemiology*	Case series	2017	United States	41 residents	To describe an outbreak of viral respiratory illness caused by RSV and hMPV in a LTCF among residents with a high rate of influenza vaccination. To highlight infection prevention challenges in a LTCF.	Among residents, 6 cases of RSV, 7 cases of hMPV and 1 case of influenza detected in the outbreak.
Uršiè *et al.*, ((8))	Viral respiratory infections in a nursing home: a six-month prospective study	*BMC Infectious Diseases*	Prospective study	2016	Slovenia	90 residents, 42 staff	To assess and compare the incidence of acute respiratory illness in nursing home residents and staff, to identify viruses involved in acute respiratory infection, and to correlate viral etiology with clinical manifestations of acute respiratory infection.	Five RSV cases detected among residents leading to 5 lower respiratory tract infections due to RSV. Zero RSV cases were detected among staff.

Abbreviations: hMPV, human metapneumovirus; LTCF, long-term care facility; RSV, respiratory syncytial virus

### Synthesis of results

The average age of the resident study population was reported in three articles (81.5, 85.5 and 84 years) ((8,21,22)). Average age of RSV positive residents was reported in one article (84 years) ((22)). The age range for residents was provided in three articles, with the overall age ranging from 68 to 90 years ((8,21,22)). One article provided the average age of staff members at the affected LTCH (average of 38 years; range: 35–46 years) ((8)). Gender proportion for residents who tested positive for RSV was provided in one article, where (50% (n=2/4) of RSV positive cases were male and 50% (n=2/4) were female ((22)). Gender proportion for staff was discussed in one article, where 97.6% of the LTCH staff identified as female ((8)). All outbreaks took place in LTCHs, with residents affected in each outbreak (n=4/4). All four articles discussed single-facility outbreaks of RSV. Discussion of staff was presented briefly in one article, in which no staff cases were reported during this RSV outbreak ((8)).

Comorbidities among residents were discussed in all four articles. In two articles, various forms of dementia were identified as a common comorbidity among the overall resident study population; however, identification of RSV-positive cases with attributable comorbidities was not available ((8,23)). In one study, no comorbidities were identified among RSV-positive cases who developed pneumonia, and comorbidities for the remaining study population were not discussed ((21)). Comorbidities reported among those who were RSV positive included respiratory allergy, hypertension, diabetes mellitus, kidney dysfunction, heart failure and frailty ((22)).

Resident vaccine status was discussed in one article, where a large proportion of residents at the LTCH were vaccinated against influenza; however, no resident vaccination rate was provided ((23)). No article discussed staff vaccine status.

Exposure source was not confirmed in any article. One article identified the possibility of pathogen introduction to the facility via infected visitors or the childcare centre/intergenerational program that operates within the facility ((22)). Presenteeism, which occurs when an employee attends work despite the presence of an illness that might prevent them from functioning fully while at work ((24)), might have been associated with RSV exposure among residents in two outbreaks (22,23).

Outbreak duration was discussed in three articles, with an average of 17 days and a range of 13 to approximately 21 days ((8,22,23)). None of the articles discussed the criteria that were used by the LTCH to determine when an outbreak was declared over.

One article reported an outbreak definition that was used by either researchers or the LTCH during their respective outbreak investigations, which required at least two cases of acute respiratory infection with identification within five days in the same unit and with laboratory confirmation of infection with the same virus ((8)). One article provided a case definition that was based on clinical manifestations of symptoms of respiratory pathogen infection; however, these identified cases also underwent testing to identify the infectious agent ((23)).

Respiratory syncytial virus cases were identified in each outbreak and ranged from four to seven resident cases per outbreak. The attack rate, defined as the proportion of a population exposed to RSV who then developed symptoms of RSV infection and tested positive for RSV, ranged from 12% to 38% for residents. In three articles, insufficient information was provided about the number of staff members at risk of infection with RSV. One article identified the number of respiratory infections during a mixed outbreak that were not due to RSV, where eight of 14 infections (57%) were due to a pathogen other than RSV, specifically human metapneumovirus (hMPV) or influenza ((23)). In three studies, all cases identified in the RSV-attributable outbreaks were due to RSV infection among residents, and no other pathogens were detected in the associated outbreaks ((8,21,22)).

Respiratory syncytial virus subtype was reported in three articles in which RSV-B was identified ((21–23)). One article did not discuss the subtype of RSV detected in the outbreak ((8)).

Information regarding co-infection was provided by two articles. One article identified one co-infection in a resident who tested positive for both RSV and hMPV ((23)). In contrast, another included article did not identify any co-infections during the RSV outbreak ((8)). Co-infections among residents infected with RSV were not discussed in two articles ((21,22)).

Symptom severity ranged from mild cold-like symptoms to more severe manifestations, including pneumonia and lower respiratory tract infection (LRTI) attributable to RSV infection. Clinical severity for cases of RSV was reported in three articles ((8,21,22)). Of these outbreaks and among those who tested positive for RSV, pneumonia was reported in two articles ((8,21)). One article reported four RSV-positive cases (n=4/4; 100%) who developed acute respiratory infection with mild cold-like symptoms ((22)). One article reported the development of pneumonia in 10 residents; however, symptoms of RSV-positive cases were not distinguishable from symptoms experienced by those who tested positive for hMPV or influenza ((23)). Clinical symptom severity for RSV cases could not be distinguished from symptoms of non-RSV infections in one article ((23)). Symptom severity among staff was excluded from three articles and was not applicable in one study because no staff cases were detected (8).

No hospitalizations due to RSV were reported in two articles ((21,22)). Information regarding resident hospitalization was not provided in two articles ((8,23)). Staff hospitalizations were not discussed in three articles ((21-23)) and one article reported no RSV cases amongst staff ((8)).

Information about resident deaths due to RSV was reported in three articles ((8,21,22)). In one article, one out of five resident RSV cases confirmed by diagnostic testing died (case fatality rate: 20%) ((8)), whereas in two articles, no case fatalities were reported and all affected residents recovered ((21,22)). Staff mortality information due to RSV was not provided in three articles ((21,23)). In one article, information regarding staff mortality was not relevant since zero staff RSV cases were detected ((8)).

Two articles discussed outbreak mitigation measures in affected LTCHs (**Table 3**) ((22,23)). In both outbreaks, cohorting of infected residents occurred, which is an effective outbreak mitigation method of separating infected and uninfected individuals during an outbreak (25). One facility struggled to cohort staff members and infected residents due to the large number of sick and absent staff ((23)). One facility reported the LTCH’s architectural layout enabled cohorting ((22)). One LTCH implemented infection prevention and control measures outlined in The Netherland’s infection prevention working group guidelines ((22)), though it is unknown which of these measures were implemented within the affected LTCH.

**Table 3:** Main findings of each included study

Author, citation	Outbreak setting	Outbreak type	Pathogen(s) detected	Pathogen detection method	Outbreak duration (days)	Residents with confirmed RSV infection (n)	Staff infected and confirmed by testing (n)	Attack rate	Co-infection(s)	Hospitalization(s) due to RSV	Death(s) due to RSV	Symptom severity	RSV subtype detected	Outbreak mitigation measures implemented	Hypothesized exposure source
Doi *et al.*, ((21))	1 LTCH; most infections contained to 2^nd^ floor	RSV	RSV	RT-PCR testing	Unknown	7	Unknown	24	Unknown	0	0	5 residents with pneumonia; 4 of 7 that tested positive for RSV presented with pneumonia and acute wheezing	RSV-B	Unknown	Unknown
Meijer *et al.*, ((22))	1 LTCH; limited to mostly 1 unit	RSV	RSV	Unknown	21^a^	4	Unknown	38	Unknown	0	0	4 diagnosed with acute respirator infection and common cold; mild symptoms	RSV-B	Followed nursing home-specific guidelines for infection prevention and control; outbreak spread mitigated due to cohorting and isolation of infected and directly exposed residents	Actual exposure source unknown; hypothesized exposure from presenteeism, sick visitors, intergenerational geriatric remotivation
Spires *et al.*, ((23))	1 LTCH; spread across 2 locked units	Mixed	RSV, hMPV, influenza	RT-PCR testing	16	6	Reported sick staff members, pathogen involved not specified	15	1	Unknown	Unknown	15 residents transferred to acute care; 10 diagnosed with pneumonia and 5 deaths^b^	RSV-B	Cohorting of infected residents into private rooms or shared rooms with another case; placement of case residents into droplet; contact precautions (e.g., limitations on travel outside patient room and use of personal protective equipment); messaging to staff to avoid presenteeism; emphasis on staff hand hygiene and proper respiratory etiquette; daily leadership meetings; cessation of unit-based group activities; visitor restrictions; closure of unit to new admissions	Actual exposure source unknown; presenteeism discussed as potential source
Uršiè *et al.*, ((8))	1 LTCH	RSV	RSV	PCR testing	13	5	0	12	0	Unknown	1	All developed lower respiratory tract infection; pneumonia also reported	Unknown	Unknown	Unknown

Abbreviations: hMPV, human metapneumovirus; LTCH, long-term care home; PCR, polymerase chain reaction; RSV, human respiratory syncytial virus; RT-PCR, reverse transcription polymerase chain reaction

^a^ This outbreak was reported to have a duration of approximately three weeks; duration was converted to approximately 21 days to keep the unit (days) for this outcome consistent across evidence sources

^b^ Could not distinguish which outcomes were due to RSV, hMPV or influenza

## Discussion

### Summary of evidence

This review highlights the lack of available evidence pertaining to RSV outbreak burden during the study period and a large knowledge gap pertaining to the burden of RSV outbreaks among LTCH staff. Symptom severity ranged from mild cold-like symptoms to pneumonia, LRTI and death. The range and severity of symptoms among residents align with what has been previously reported. Severe respiratory symptoms in older adults in LTCH may be more likely to occur due to age-associated immunity impairments, presence of comorbid conditions, such as diabetes mellitus and chronic obstructive pulmonary disease, living conditions within LTCHs, and existing prevalence of pneumonia and LRTI among older adults ((14,19,26,27)). Older adults in LTC, particularly those prone to frailty, may be at even greater risk of severe complications due to RSV infection ((19,28)). Additionally, a large proportion of residents developed LRTI attributable to RSV, which aligns with existing literature as RSV is a major cause of LRTI ((29)).

Considering the lack of available vaccination information for both staff and residents, data on routine vaccinations in older adults could be improved. Improved collection of routine vaccination data for multiple respiratory pathogens, including influenza, severe acute respiratory syndrome coronavirus 2 and pneumococcal infections could improve mitigation and allow for intervention research on new vaccines in LTCH and at-risk populations.

Some possible exposure sources included presenteeism, the presence of intergenerational programs within the LTCH and the introduction of RSV into the facility by visitors or volunteers ((22–24)). Further research to understand the relative impact of the introduction of RSV into LTCHs and the identification of common exposure sources or transmission routes associated with RSV outbreaks in LTCHs may be useful to inform more effective outbreak management ((30–32)). Cohorting staff and residents in affected LTCH units as part of outbreak management was an identified challenge. Additional research pertaining to the effectiveness of cohorting methods and other outbreak mitigation measures may benefit a LTCH’s ability to strategically prepare outbreak management plans.

This scoping review meets the objective to understand the current state of evidence regarding RSV outbreak burden data among residents and staff in LTCHs. Currently, the evidence base is scarce, so the integration of RSV surveillance and the publication of these data are important to better characterize the epidemiology of RSV outbreaks in LTCHs and to inform public health interventions to prevent and respond to RSV outbreaks among LTC populations. However, although sparse, the evidence in this review shows that RSV outbreaks have occurred in LTCHs and required enhanced measures to control—often over many days (13–21 days). Respiratory syncytial virus outbreaks in LTC can cause morbidity and mortality among residents. In some cases, symptoms are mild and self-limiting, while in others, attack rates and severe outcomes including hospitalization, pneumonia and death are documented.

## Strengths and limitations

This review highlights gaps in the knowledge base rather than generating novel ideas, which is a critical part of the exploratory research process. The application of broad inclusion criteria enabled a sensitive literature search, so it is likely that this article provides an accurate picture of currently available published evidence. Article screening and abstraction were conducted by a single reviewer, which could increase the risk of introducing errors and biases. Lastly, a grey literature search was not conducted due to time constraints, which might have excluded some relevant data sources since surveillance reports are often not published in peer-reviewed journals.

## Conclusion

This scoping review highlights a lack of published, peer-reviewed evidence pertaining to RSV outbreak burden in LTC settings. There is a paucity of available evidence that describes RSV outbreak burden among residents and particularly among staff members. This evidence could help to inform future research and population-specific public health measures to reduce the burden of RSV outbreaks in LTCHs.

Consideration of qualitative factors, like RSV outbreaks’ impact on physical symptomatology, mental health and financial impacts or factors that might influence the risk of presenteeism might provide important evidence to inform outbreak management and response in LTCHs. Population-wide studies to describe the epidemiology of RSV outbreaks in LTCHs could also provide valuable data for public health interventions. Overall, the implementation of RSV outbreak surveillance, and its integration with surveillance of other respiratory pathogens in LTC, could enable better characterization of the susceptibility, transmissibility and virulence of RSV and other respiratory pathogens in LTCHs. The results of this scoping review also highlight the need for public health organizations to publish findings from outbreak investigations, so this evidence can be used to inform public health policy, practice and decision making to prevent and respond to RSV outbreaks in LTC.

## Appendix

**Table A1:** Sample search strategy utilized by the Health Canada Library Database(s): Ovid MEDLINE(R) ALL 1946 to February 14, 2023

#	Searches	Results
1	Respiratory Syncytial Virus Infections/	8,468
2	(respiratory syncytial vir* or respiratory syncytial pneumov* or RSV or hrsv).tw,kf,kw.	20,982
3	1 or 2 [RSV]	21,627
4	exp *Aged/ or *Geriatrics/	53,958
5	(((old* or aging) adj (adult* or woman or women or man or men or people? or person? or resident?)) or elder? or geriatric* or aging or ageing or senior? or retiree* or retired or pensioner* or “over 65” or “over 80” or baby boomer? or babyboomer? or silent generation or septuagenarian? or octogenarian? or nonagenarian? or centenarian? or geronol* or ((“65” or “66” or “67” or “68” or “69” or 7# or 8# or 9# or “100”) adj year*)).ti,kf. or (((older or aging) adj (adult* or woman or women or man or men or people? or person? or resident?)) or elder? or geriatric* or aging or ageing or senior? or retiree* or retired or pensioner* or “over 65” or “over 80” or baby boomer? or babyboomer? or silent generation or septuagenarian? or octogenarian? or nonagenarian? or centenarian? or geronol* or ((“65” or “66” or “67” or “68” or “69” or 7# or 8# or 9# or “100”) adj year*)).ab. /freq=2	461,347
6	4 or 5 [Older Adults]	481,298
7	long-term care/ or exp nursing homes/	66,904
8	((long term or extend* or continu* or advance* or chronic* or aged) adj3 care).tw,kf.	91,739
9	(((nursing or retirement* or care) adj2 (home* or communit* or facilit*)) or assisted living* or hopsice*).tw,kf.	122,066
10	or/7-9 [Long Term Care Homes]	218,119
11	exp “quality of life”/ or exp morbidity/ or exp mortality/ or hospitalization/ or exp critical care/ or “severity of illness index”/ or “length of stay”/ or “cost of illness”/ or Respiratory Tract Infections/ or exp bronchitis/ or Healthcare-Associated Pneumonia/ or Multiple Organ Failure/ or Sepsis/	1,840,212
	(mortalit* or morbidit* or death? or die or died).tw,kw,kf.	2,126,484
13	(“quality of life” or “quality adjusted life year*” or qaly or “disability adjusted life year*” or daly).tw,kw,kf.	371,153
14	((health* or wellness or disease* or disorder* or illness* or condition*) adj3 burden*).tw,kf.	74,734
15	(Burden* or Proportion or Case fatality rate* or case fatality ratio* or CFR or Attack rate* or Hospitalization* or Intensive care unit* or ICU or incidence* or duration* or span or timespan or period of time or length or Respirator* failure* or respiratory tract infection* or URTI or LRTI or Bronchi* or Pneumonia or Multi* organ failure* or Sepsis or septic* or Fever* or outbreak*).tw,kw,kf.	3,845,127
16	or/11-15	6,297,775
17	exp australia/ or austria/ or exp baltic states/ or exp belgium/ or exp canada/ or chile/ or czech republic/ or exp “scandinavian and nordic countries”/ or exp france/ or exp germany/ or greece/ or hungary/ or exp ireland/ or israel/ or exp italy/ or exp japan/ or exp republic of korea/ or luxembourg/ or mexico/ or exp netherlands/ or exp new zealand/ or poland/ or exp portugal/ or slovakia/ or slovenia/ or exp spain/ or exp switzerland/ or turkey/ or exp united kingdom/ or exp united states/ or (australia* or new south wales or queensland or tasmania or victoria or sydney or melbourne or brisbane or austria* or vienna or viennese* or belgium* or belgian* or brussels or flemish* or canad* or ottawa* or british columbia* or colombie britannique* or vancouver* or alberta* or edmonton* or calgar* or saskatchewan* or regina* or saskatoon* or manitoba* or winnipeg* or ontari* or toronto* or quebec* or montreal* or new brunswick* or nouveau brunswick* or fredericton* or nova scotia* or nouvelle ecosse* or halifax* or haligonian* or prince edward island* or ile du prince edouard* or pei or charlottetown* or newfoundland* or terre neuve* or labrador* or nfld or yukon* or whitehorse* or northwest territor* or territoires du nord ouest* or nwt or yellowknife* or nunavut* or iqaluit* or chile* or santiago or czech* or prague or denmark* or danish or dane* or faroe* or copenhagen or estonia* or tallinn or finland* or finnish* or helsinki* or france* or french* or paris* or marseille or lyon or lille or nice or toulouse or bordeaux or german* or deutschland* or berlin* or hamburg or munich or cologne or frankfurt or stuttgart or dusseldorf or greece* or hellenic* or greek* or athens or macedonia* or hungary* or hungarian* or budapest or iceland* or reykjavik or ireland* or irish* or dublin* or israel* or jerusalem or tel aviv or italy or italian* or rome or milan or naples or turin or sicily or japan* or tokyo or yokohama or osaka or nagoya or sapporo or kobe or kyoto or korea* or seoul or busan or daegu or daejeon or gwangju or incheon or ulsan or latvia* or riga or lithuania* or vilnius or luxembourg* or netherland* or holland* or dutch* or amsterdam or rotterdam or hague or new zealand* or aotearoa or wellington or auckland or maori or mexic* or norway* or norwegian* or oslo or poland* or polish or warsaw or krakow or wroclaw or lodz or portug* or lisbon or slovak* or bratislava or slovenia* or slovene* or ljubljana or spain* or spanish* or spaniard* or madrid or barcelona or catalonia* or valencia* or seville or zaragoza or malaga or basque or scandinavia* or sweden or swedish or swede* or stockholm or switzerland* or swiss* or zurich or geneva or bern or turkey or turkish or istanbul or constantinople or britain* or british* or united kingdom* or scotland* or scottish or wales* or welsh or england* or belfast or london or manchester or glasgow or birmingham or leeds or bradford or liverpool or alabama* or alaska* or arizona* or arkansas* or california* or colorado* or connecticut* or delaware* or florida* or georgia* or hawaii* or idaho* or illinois* or indiana* or iowa* or kansas* or kentucky* or louisiana* or maine* or maryland* or massachusetts* or michigan* or minnesota* or mississippi* or missouri* or montana* or nebraska* or nevada* or new hampshire* or new jersey* or new mexico* or new york* or north carolina* or north dakota* or ohio* or oklahoma* or oregon* or pennsylvania* or rhode island* or south carolina* or south dakota* or tennessee* or texas* or utah* or vermont* or virginia* or washington* or west virginia* or wisconsin* or wyoming* or montgomery* or juneau* or anchorage* or phoenix* or little rock* or sacramento* or los angeles* or san diego* or san francisco* or denver* or hartford* or dover* or tallahassee* or miami* or orlando* or atlanta* or honolulu* or boise* or springfield* or chicago* or des moines* or topeka* or frankfort* or baton rouge* or new orleans* or augusta* or annapolis* or boston* or lansing* or detroit* or st?paul* or jackson* or jefferson city* or helena* or lincoln* or carson city* or reno* or las vegas* or concord* or trenton* or santa fe* or albany* or raleigh* or bismarck* or columbus* or oklahoma city* or salem* or harrisburg* or providence* or columbia* or peirre* or nashville* or austin* or dallas* or salt lake city* or montpelier* or richmond* or olympia* or seattle* or charleston* or madison* or cheyenne* or district of columbia* or usa or united states or europ* or north america*).tw,kf.	5,302,035
18	3 and (6 or 10) and 16 and 17	152
19	limit 18 to yr=2010-2020	55
20	limit 19 to english	55
21	limit 19 to french	1
22	20 or 21	55

## References

[1] Belongia EA, King JP, Kieke BA, Pluta J, Al-Hilli A, Meece JK, Shinde V. Clinical Features, Severity, and Incidence of RSV Illness During 12 Consecutive Seasons in a Community Cohort of Adults ≥60 Years Old. Open Forum Infect Dis 2018;5(12):ofy316. 10.1093/ofid/ofy316 30619907 PMC6306566

[2] Hall CB, Simőes EA, Anderson LJ. Clinical and epidemiologic features of respiratory syncytial virus. Curr Top Microbiol Immunol 2013;372:39–57. 10.1007/978-3-642-38919-1_2 24362683

[3] Tin Tin Htar M, Yerramalla MS, Moïsi JC, Swerdlow DL. The burden of respiratory syncytial virus in adults: a systematic review and meta-analysis. Epidemiol Infect 2020;148:e48. 10.1017/S0950268820000400 32052719 PMC7078512

[4] Branche AR, Falsey AR. Respiratory syncytial virus infection in older adults: an under-recognized problem. Drugs Aging 2015 Apr;32(4):261–9. 10.1007/s40266-015-0258-9 25851217

[5] Nguyen-Van-Tam JS, O’Leary M, Martin ET, Heijnen E, Callendret B, Fleischhackl R, Comeaux C, Tran TM, Weber K. Burden of respiratory syncytial virus infection in older and high-risk adults: a systematic review and meta-analysis of the evidence from developed countries. Eur Respir Rev 2022;31(166):220105. 10.1183/16000617.0105-2022 36384703 PMC9724807

[6] Savic M, Penders Y, Shi T, Branche A, Pirçon JY. Respiratory syncytial virus disease burden in adults aged 60 years and older in high-income countries: A systematic literature review and meta-analysis. Influenza Other Respir Viruses 2023;17(1):e13031. 10.1111/irv.13031 36369772 PMC9835463

[7] Jansen AG, Sanders EA, Hoes AW, van Loon AM, Hak E. Influenza- and respiratory syncytial virus-associated mortality and hospitalisations. Eur Respir J 2007;30(6):1158–66. 10.1183/09031936.00034407 17715167

[8] Uršič T, Miksić NG, Lusa L, Strle F, Petrovec M. Viral respiratory infections in a nursing home: a six-month prospective study. BMC Infect Dis 2016;16(1):637. 10.1186/s12879-016-1962-8 27814689 PMC5097393

[9] Anderson EJ, Hussaini L, Bristow L, Tippett A, Gibson T, Hart M, Salazar L, Gaffney M, Kanayo Benyeogor I, Cheng A, Drobeniuc A, Traenkner J, Fayad D, Washington W, Emerson L, Schwartz N, Greaves K, Todd S, Stanley C, Bechnak A, Bou Chaaya R, Al-Husien Z, Deovic R, Winston J, Rafi Ahmed D, Li W, Singh A, Spencer JE, Nuchinsky A, Zaks KM, Nesheim W, Stephens K, Swerdlow DL, Hubler R, Agosti Y, Munye M, Jadhao S, Ha B, McCracken C, Kraft C, Rostad CA, Kao C, Lopman B, Yildirim I, Anderson L, Rouphael N, Rouphael N. Burden of Respiratory Syncytial Virus (RSV) Infection Among Hospitalized Older Adults and Those with Underlying Chronic Obstructive Pulmonary Disease (COPD) or Congestive Heart Failure (CHF). Open Forum Infect Dis 2314;6 Suppl 2:S793–4. 10.1093/ofid/ofz360.1992 10.1093/ofid/ofz360.1992

[10] Tseng HF, Sy LS, Ackerson B, Fischetti C, Slezak J, Luo Y, Solano Z, Chen S, Shinde V. Morbidity, and Short- and Intermediate-term Mortality, in Adults ≥60 Years Hospitalized with Respiratory Syncytial Virus Infection vs. Seasonal Influenza Virus Infection. Open Forum Infect Dis 2017;4 Suppl 1:S318–9. 10.1093/ofid/ofx163.747

[11] Widmer K, Zhu Y, Williams JV, Griffin MR, Edwards KM, Talbot HK. Rates of hospitalizations for respiratory syncytial virus, human metapneumovirus, and influenza virus in older adults. J Infect Dis 2012;206(1):56–62. 10.1093/infdis/jis309 22529314 PMC3415933

[12] Ontario Ministry of Health and Long-Term Care. Control of Respiratory Infection Outbreaks in Long-Term Care Homes, 2018. Toronto, ON: MOH; 2018. https://files.ontario.ca/moh-ophs-ref-control-respiratory-infection-outbreaks-ltc-homes-2018-en.pdf

[13] Nazareno AL, Muscatello DJ, Turner RM, Wood JG, Moore HC, Newall AT. Modelled estimates of hospitalisations attributable to respiratory syncytial virus and influenza in Australia, 2009-2017. Influenza Other Respir Viruses 2022;16(6):1082–90. 10.1111/irv.13003 35775106 PMC9530581

[14] Smith PW, Bennett G, Bradley S, Drinka P, Lautenbach E, Marx J, Mody L, Nicolle L, Stevenson K. SHEA; APIC. SHEA/APIC Guideline: Infection Prevention and Control in the Long-Term Care Facility. Infect Control Hosp Epidemiol 2008;29(9):785–814. 10.1086/592416 18767983 PMC3319407

[15] Utsumi M, Makimoto K, Quroshi N, Ashida N. Types of infectious outbreaks and their impact in elderly care facilities: a review of the literature. Age Ageing 2010;39(3):299–305. 10.1093/ageing/afq029 20332371

[16] Katz PR. An international perspective on long term care: focus on nursing homes. J Am Med Dir Assoc 2011;12(7):487–492.e1. 10.1016/j.jamda.2011.01.017 21450252

[17] Spetz J, Trupin L, Bates T, Coffman JM. Future Demand For Long-Term Care Workers Will Be Influenced By Demographic And Utilization Changes. Health Aff (Millwood) 2015;34(6):936–45. 10.1377/hlthaff.2015.0005 26056198

[18] Statistics Canada. A portrait of Canada’s growing population aged 85 and older from the 2021 Census. Ottawa, ON: StatCan; 2022. https://www12.statcan.gc.ca/census-recensement/2021/as-sa/98-200-X/2021004/98-200-X2021004-eng.cfm

[19] Juthani-Mehta M, Quagliarello V. Infections in Long-Term Care Facilities. In: Scheld WM, Grayson ML, Hughes JM, editors. Emerging Infections 9. Wiley Online Books; 2010. p. 287–303. 10.1128/9781555816803.ch15 10.1128/9781555816803.ch15

[20] Page MJ, McKenzie JE, Bossuyt PM, Boutron I, Hoffmann TC, Mulrow CD, Shamseer L, Tetzlaff JM, Akl EA, Brennan SE, Chou R, Glanville J, Grimshaw JM, Hróbjartsson A, Lalu MM, Li T, Loder EW, Mayo-Wilson E, McDonald S, McGuinness LA, Stewart LA, Thomas J, Tricco AC, Welch VA, Whiting P, Moher D. The PRISMA 2020 statement: an updated guideline for reporting systematic reviews. BMJ 2021;372(71):n71. 10.1136/bmj.n71 33782057 PMC8005924

[21] Doi I, Nagata N, Tsukagoshi H, Komori H, Motoya T, Watanabe M, Keta T, Kawakami M, Tsukano T, Honda M, Ishioka T, Takeda M, Ryo A, Kuroda M, Oishi K, Kimura H. An outbreak of acute respiratory infections due to human respiratory syncytial virus in a nursing home for the elderly in Ibaraki, Japan, 2014. Jpn J Infect Dis 2014;67(4):326–8. 10.7883/yoken.67.326 25056086

[22] Meijer A, Overduin P, Hommel D, van Rijnsoever-Greven Y, Haenen A, Veldman-Ariesen MJ. Outbreak of respiratory syncytial virus infections in a nursing home and possible sources of introduction: the Netherlands, winter 2012/2013. J Am Geriatr Soc 2013;61(12):2230–1. 10.1111/jgs.12565 24329823

[23] Spires SS, Talbot HK, Pope CA, Talbot TR. Paramyxovirus Outbreak in a Long-Term Care Facility: The Challenges of Implementing Infection Control Practices in a Congregate Setting. Infect Control Hosp Epidemiol 2017;38(4):399–404. 10.1017/ice.2016.316 28065183

[24] Widera E, Chang A, Chen HL. Presenteeism: a public health hazard. J Gen Intern Med 2010;25(11):1244–7. 10.1007/s11606-010-1422-x 20549378 PMC2947637

[25] Rosenberger LH, Riccio LM, Campbell KT, Politano AD, Sawyer RG. Quarantine, isolation, and cohorting: from cholera to Klebsiella. Surg Infect (Larchmt) 2012;13(2):69–73. 10.1089/sur.2011.067 22472002 PMC4845677

[26] Falsey AR. Respiratory syncytial virus infection in adults. Semin Respir Crit Care Med 2007;28(2):171–81. 10.1055/s-2007-976489 17458771

[27] Stephens LM, Varga SM. Considerations for a Respiratory Syncytial Virus Vaccine Targeting an Elderly Population. Vaccines (Basel) 2021;9(6):624. 10.3390/vaccines9060624 34207770 PMC8228432

[28] Bosco E, van Aalst R, McConeghy KW, Silva J, Moyo P, Eliot MN, Chit A, Gravenstein S, Zullo AR. Estimated Cardiorespiratory Hospitalizations Attributable to Influenza and Respiratory Syncytial Virus Among Long-term Care Facility Residents. JAMA Netw Open 2021;4(6):e2111806. 10.1001/jamanetworkopen.2021.11806 34106266 PMC8190624

[29] Mosscrop LG, Williams TC, Tregoning JS. Respiratory syncytial virus after the SARS-CoV-2 pandemic - what next? Nat Rev Immunol 2022;22(10):589–90. 10.1038/s41577-022-00764-7 35831610 PMC9281204

[30] National Academies of Sciences, Engineering, and Medicine. Global Health Risk Framework. Washington, DC: National Academies Press; 2016. https://nap.nationalacademies.org/catalog/21856/global-health-risk-framework-resilient-and-sustainable-health-systems-to 26962612

[31] Chen X, Chong WF, Feng R, Zhang L. Pandemic risk management: resources contingency planning and allocation. Insur Math Econ 2021;101:359–83. 10.1016/j.insmatheco.2021.08.001 34803199 PMC8593845

[32] Hempel S, Burke RV, Hochman M, Thompson G, Brothers A, Shin J, Motala A, Larkin J, Ringel J. Resource Allocation and Pandemic Response: An Evidence Synthesis to Inform Decision-Making. Agency for Healthcare Research and Quality (US); 2020. Report No.: 20(21)-EHC027. PMID:33054151 33054151

